# Integrated Gas Sensing System of SWCNT and Cellulose Polymer Concentrator for Benzene, Toluene, and Xylenes

**DOI:** 10.3390/s16020183

**Published:** 2016-02-02

**Authors:** Jisun Im, Elizabeth S. Sterner, Timothy M. Swager

**Affiliations:** Department of Chemistry, Massachusetts Institute of Technology, Cambridge, MA 02139, USA; jisun_im@mit.edu (J.I.); esterner@mit.edu (E.S.S.)

**Keywords:** cellulose, SWCNT, gas sensor, benzene, concentrator

## Abstract

An integrated cellulose polymer concentrator/single-walled carbon nanotube (SWCNT) sensing system is demonstrated to detect benzene, toluene, and xylenes (BTX) vapors. The sensing system consists of functionalized cellulose as a selective concentrator disposed directly on top of a conductive SWCNT sensing layer. Functionalized cellulose concentrator (top layer) selectively adsorbs the target analyte and delivers the concentrated analyte as near as possible to the SWCNT sensing layer (bottom layer), which enables the simultaneous concentrating and sensing within a few seconds. The selectivity can be achieved by functionalizing cellulose acetate with a pentafluorophenylacetyl selector that interacts strongly with the target BTX analytes. A new design of the integrated cellulose concentrator/SWCNT sensing system allows high sensitivity with limits of detection for benzene, toluene, and *m*-xylene vapors of 55 ppm, 19 ppm, and 14 ppm, respectively, selectivity, and fast responses (<10 s to reach equilibrium), exhibiting the potential ability for on-site, real-time sensing applications. The sensing mechanism involves the selective adsorption of analytes in the concentrator film, which in turn mediates changes in the electronic potentials at the polymer-SWCNT interface and potentially changes in the tunneling barriers between nanotubes.

## 1. Introduction

Benzene, toluene, and xylenes (BTX) are important chemicals as starting and intermediate materials for a wide range of products and are produced largely from petroleum, e.g., pyrolysis gasoline and reformate. BTX are, however, toxic and benzene is classified as a carcinogen [1]. Therefore, facile methods to monitor BTX in chemical and petrochemical industries and refineries are essential for safety and to protect the environment as well as process and quality control.

Currently available monitoring systems for BTX are based mainly on gas chromatography (GC) coupled with mass spectrometer (MS) and flame ionization detection (FID) [2,3,4,5,6,7]. The detection limits of GC/MS or GC/FID techniques can be down to low parts-per-billion (ppb), but there are limitations for real time, on-site analysis of BTX traces due to relatively long sampling, preconcentrating, and transferring steps prior to detection. Furthermore, operation and analysis expertise are required for the detection. Consequently, a portable and easily operable sensor for on-site, real time field monitoring with high sensitivity, selectivity, and fast response time is desirable.

Optical sensors [8,9,10,11] based on infrared and ultraviolet spectroscopies and optical waveguides for on-site field BTX detection have been reported to be portable and show sub-parts-per-million (ppm) detection limits. In most cases, a preconcentration step prior to sensing is necessary to detect sub-ppm level of BTX gases. A preconcentrator typically consisting of polymeric or carbon-based absorbents collects analytes, and the concentrated analytes are then delivered to a sensing unit by thermal desorption [12,13,14,15]. The preconcentration step takes from tens of minutes to several hours, and a thermal desorption system requires high power consumption, which makes a system challenging for portable and on-site field sensing applications.

**Figure 1:**
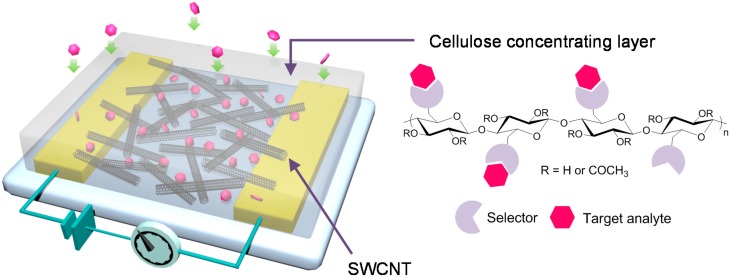
Schematic diagram of the integrated polymer concentrator/single-walled carbon nanotubes (SWCNT) sensing system: cellulose concentrating layer absorbs, concentrates, and delivers analytes as near as possible to the SWCNT sensing layer within a few seconds.

Here, we present an integrated polymer concentrator/chemiresistive sensing system that can detect tens of ppm of BTX gases within a few seconds at room temperature without a long preconcentrating step. The system consists of a polymer concentrating layer disposed directly on top of a conductive sensing layer. Single-walled carbon nanotubes (SWCNTs) are used as a conductive sensing material in this study. Carbon nanotubes (single or bundle) and their composites have proven effective in sensing applications owing to their sensitivity (e.g., change in electronic properties) towards chemical environments [16,17,18,19,20,21]. However, their selectivity issue towards the specific target still remains challenging. In attempting to impart high sensitivity and selectivity to the SWCNT sensor, we employed functionalized cellulose acetate (CA) as a polymer concentrator overcoated directly on top of SWCNTs (Figure 1). The functionalized cellulose concentrator not only provides the selectivity by adsorbing the analyte of interest selectively, but also it keeps the concentrated analyte atmosphere at the interface between SWCNTs and cellulose concentrating layer to improve the sensitivity of the system. In addition, this system does not require the solution processing step to modify SWCNTs with selectors for the target analyte either covalently or noncovalently. Instead, the selectivity can be achieved by overcoating the functionalized cellulose acetate with selectors that interact selectively with the target analyte. A new design of our system allows concentrating and sensing simultaneously within a few seconds, exhibiting the potential ability for on-site, real-time sensing applications.

## 2. Experimental Section

### 2.1. Materials

Chemicals were purchased from Sigma-Aldrich (Natick, MA, USA), Alfa Aesar (Haverhill, MA, USA), and Macron Chemicals (Center Valley, PA, USA) and used as received except that tetrahydrofuran (THF) was dried by distillation. Deuterated solvents for NMR were obtained from Cambridge Isotope Laboratories, Inc. (Tewksbury, MA, USA). Cellulose acetate (CA-320S NF/EP) with the acetyl content of 31.9 wt% was kindly provided from Eastman (Kingsport, TN, USA). SWCNT (carbon > 90% and carbon ≥ 70% as SWCNT) was purchased from Aldrich (704113-1G). The analytes including benzene, toluene, *ortho*-, *meta*-, and *para*-xylenes, ethanol, and *n*-heptane were reagent grade and used as received.

### 2.2. Characterization

^1^H, ^13^C, and ^19^F NMR spectra were recorded on Varian Mercury (300 MHz) (Varian, Palo Alto, CA, USA) and Inova (500 MHz) NMR spectrometers (Varian, Palo Alto, CA, USA). The ^13^C NMR spectra for functionalized cellulose acetates were recorded at 60 °C. Chemical shifts are reported in parts per million (ppm) and referenced to the residual solvent resonance. High-resolution mass spectra (HRMS) were obtained on a Bruker Daltonics APEXIV 4.7 Tesla Fourier Transform Ion Cyclotron Mass Spectrometer (Bruker, Billerica, MA, USA) at the MIT Department of Chemistry Instrumentation Facility (DCIF). Thermal stabilities of functionalized cellulose acetates were studied using a thermogravimetric analysis (TGA, Discovery TGA from TA Instruments, New Castle, DE, USA). Weight loss was monitored by heating a sample from 30 °C to 600 °C at a heating rate of 20 °C/min under air atmosphere. The number average molecular weight (M_n_), weight average molecular weight (M_w_), and polydispersity index (PDI) were obtained from a gel permeation chromatography (GPC, Agilent 1100 Series, Lexington, MA, USA). THF was used as a solvent and a refractive index detector was used to obtain the molecular weights of functionalized cellulose acetates. Scanning electron microscope (SEM) images were obtained from JEOL JSM-6700F field emission SEM (Peabody, MA, USA).

### 2.3. Syntheses of Functionalized Cellulose Acetates

**Scheme 1:**
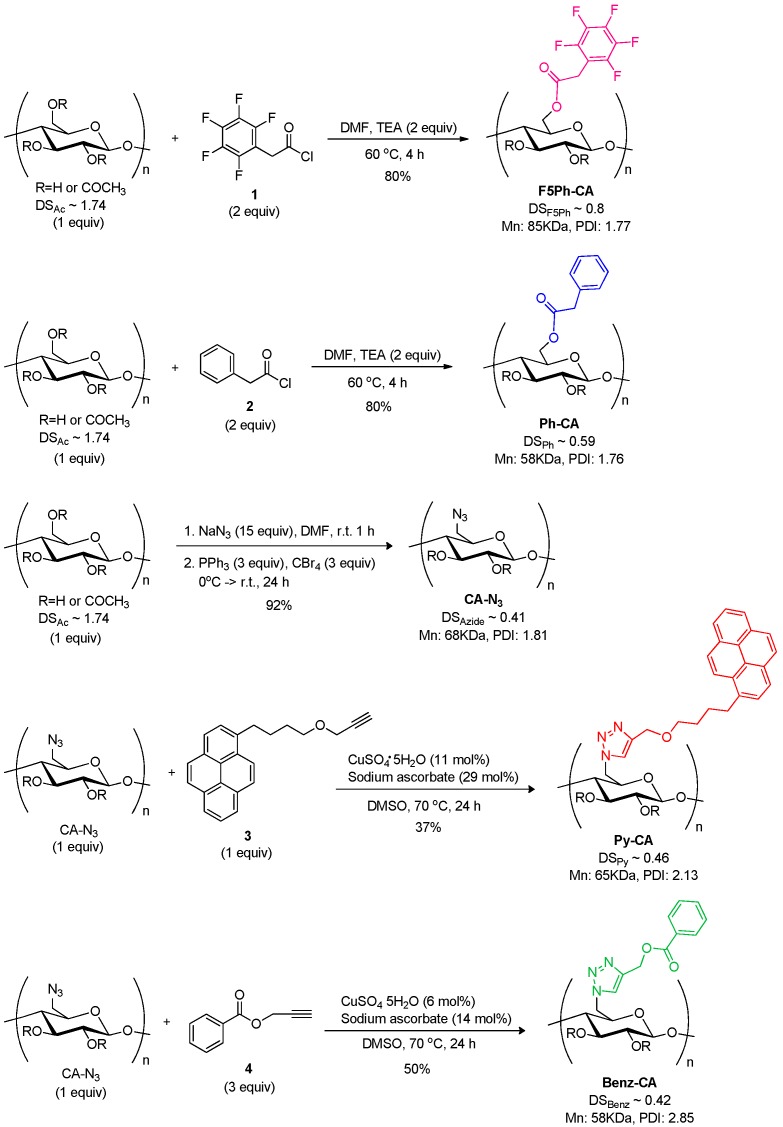
Syntheses of cellulose acetates functionalized with the selectors including 2,3,4,5,6-pentafluorophenylacetyl (F5Ph, **1**), phenylacetyl (Ph, **2**), pyrene (Py, **3**), and benzoate (Benz, **4**) groups. Pyrene and benzoate selectors were incorporated into the backbone via copper(I)-catalyzed azide-alkyne cycloaddition reaction between propargyl pyrenebutyl ether **3** and benzoate **4** and azide-functionalized cellulose acetate (CA-N_3_). The degree of substitution (DS) is determined from elemental analysis (see Supplementary Material).

**Figure 2:**
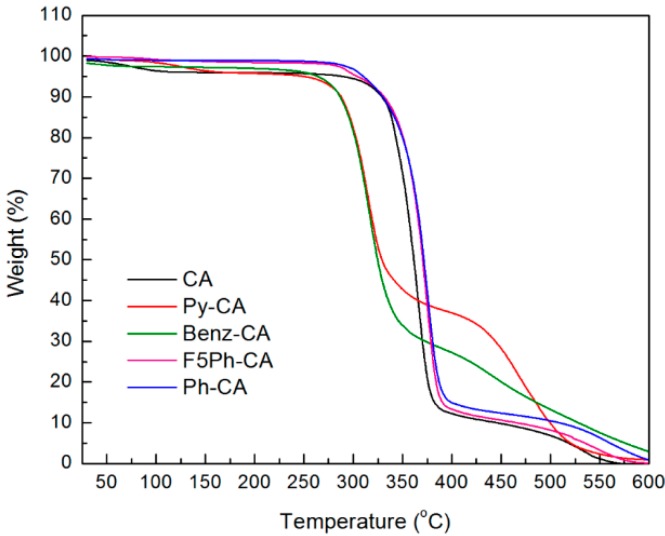
TGA curves of functionalized cellulose acetates including CA (starting material, DS_Ac_ ~ 1.74), Py-CA, Benz-CA, F5Ph-CA, and Ph-CA.

The target components-benzene, toluene, and xylenes- are aromatic hydrocarbons, and as a result, selectors with fused aromatic rings for π-π stacking or electrophilic character for electrostatic interaction are promising. Therefore, we incorporated a series of selectors including pyrene (Py), benzoate (Benz), 2,3,4,5,6-pentafluorophenylacetyl (F5Ph), and phenylacetyl (Ph) groups into the cellulose acetate backbone for the target analytes. Cellulose acetate with the acetyl content of 31.9 wt% (corresponding to the degree of substitution of acetyl groups (DS_Ac_) of 1.74) was used as a starting material. It is soluble in dimethyl sulfoxide (DMSO) and dimethylformamide (DMF), and 51% of the primary hydroxyl groups at C6 position (estimated from ^13^C NMR spectrum) can be utilized for further functionalization while keeping acetate functional groups. Scheme 1 shows the chemical structures and syntheses of cellulose acetate functionalized with 2,3,4,5,6-pentafluorophenylacetyl (F5Ph), phenylacetyl (Ph), azide (N_3_), pyrene (Py), and benzoate (Benz) groups. The detailed syntheses and characterization of the functionalized cellulose acetates and selectors (**1**, **2**, **3**, and **4** in Scheme 1) are described in the Supplementary Information (Figure S1−S6). Briefly, 2,3,4,5,6-pentafluorophenylacetyl- and phenylacetyl-functionalized cellulose acetates (F5Ph-CA and Ph-CA) were, respectively, synthesized by esterification reaction of cellulose acetates with 2,3,4,5,6-pentafluorophenylacetyl chloride **1** and phenylacetyl chloride **2** in the presence of triethylamine (TEA) at 60 °C for 4 h. The polymers were precipitated in methanol. The filtered products were further washed with methanol and dried under vacuum. The degrees of substitution of F5Ph selector (DS_F5Ph_) and Ph selector (DS_Ph_) were 0.80 and 0.59, respectively, from elemental analysis, and both F5Ph-CA and Ph-CA were soluble in acetone, THF, DMSO, and DMF. 6-Deoxy-6-azido cellulose acetate (CA-N_3_) was synthesized via one pot azidation with 92% yield by modifying the previously reported procedure [22]. Briefly, cellulose acetate was premixed with excess sodium azide (NaN_3_) in DMF at room temperature for 1 h. Triphenylphosphine (PPh_3_) was added to the solution at 0 °C, and carbon tetrabromide (CBr_4_) in DMF was then added dropwise to the solution. The mixture was allowed to warm to room temperature and stirred for 24 h. The degree of substitution of azide (DS_azide_) was calculated to be 0.41 from elemental analysis. Pyrene- and benzoate-functionalized cellulose acetates (Py-CA and Benz-CA) were synthesized via copper (I)-catalyzed azide-alkyne cycloaddition (CuAAC) reaction between propargyl pyrenebutyl ether **3** and benzoate **4** and CA-N_3_, respectively, in the presence of CuSO_4_·5H_2_O and sodium ascorbate at 70 °C for 24 h. The polymers were precipitated in methanol while stirring. The filtered polymers were further washed with H_2_O and methanol and dried under vacuum at room temperature. The degrees of substitution of pyrene (DS_Py_) and benzoate (DS_Benz_) selectors were 0.46 and 0.42 from elemental analysis, respectively, and both Py-CA and Benz-CA were soluble in DMSO and THF. The functionalized cellulose acetates were thermally stable up to 300 °C (Figure 2).

### 2.4. Quartz Crystal Microbalance Measurements

Quartz crystal microbalance (QCM) experiments were performed using Q-Sense E1 (Q-Sense, Stockholm, Sweden) to test the ability of functionalized cellulose acetates to adsorb target analytes by monitoring the frequency change when they were exposed to analyte vapors. The functionalized cellulose acetates were dropcast onto a gold-coated AT-cut quartz crystal sensor (QSX 301 Gold, Q-Sense, Stockholm, Sweden) with 5 MHz fundamental resonance frequency. 20 μg of each material from the solution of 2 mg/mL was deposited on a QCM sensor. Acetone was used as a solvent for F5Ph-CA and Ph-CA, and THF was used for Py-CA and Benz-CA. The films on QCM sensors were tested towards 500 ppm of benzene (0.47% of the saturated vapor) and toluene (1.3% of the saturated vapor) vapors which were generated from a gas generator (FlexStream^TM^ FlexBase Module, KIN-TEK Laboratories, Inc., La Marque, TX, USA) with dry nitrogen carrier gas. The concentration of vapor was calibrated by measuring mass change of an analyte after purging nitrogen gas through the analyte as a function of time at a fixed flow rate and temperature. The frequency change (the 3rd overtone, *f_3_*) of a film on a QCM sensor was measured by three cycles of exposure of a film to an analyte vapor for 1 min at 23 °C. Since the fundamental frequency is much more sensitive to the mounting of the sensor and the conditions of the sample environment than the overtones in the Q-Sense system, the 3rd overtone was monitored during experiments. The mass change (Δm) was converted from the frequency change (Δf) using Sauerbrey equation [23]:

(1) $$\Delta m=-C\frac{1}{n}\Delta f,C=\frac{t_{q}\rho_{q}}{f_{o}}$$

where *n, t_q_, ρ_q_,* and *f_o_* are the overtone, the thickness of quartz, the density of quartz, and the resonant frequency of the crystal, respectively. The mass sensitivity (C) of the crystal is 17.7 ng·cm^−2^·s^−1^ for a 5 MHz crystal.

### 2.5. Fabrication of Sensors

**Figure 3:**
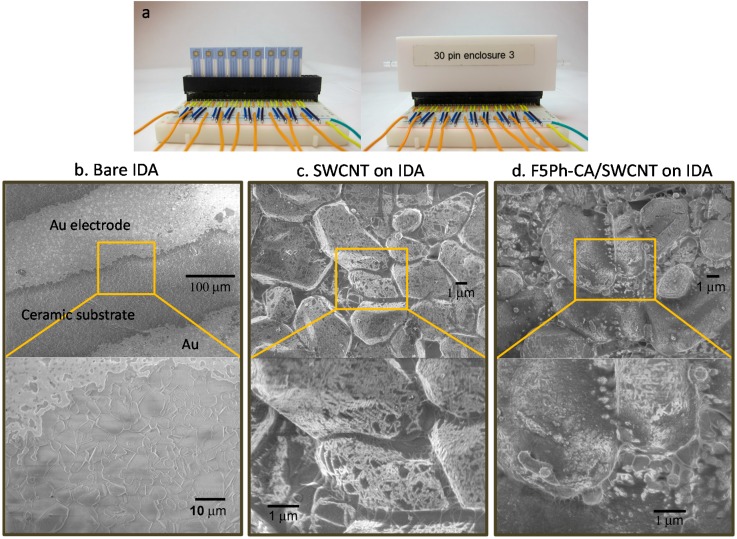
(**a**) Photographs of the array device containing 9 sensors deposited on interdigitated microelectrode array (IDA). SEM images of (**b**) bare IDA, (**c**) SWCNTs on IDA, and (**d**) F5Ph-CA/SWCNT on IDA.

The sensing system consists of two layers: the polymer concentrator layer of functionalized cellulose acetates on top of the SWCNT sensing layer. The SWCNT dispersion was prepared by sonicating the solution of SWCNT in THF with 20 μg/mL for 1 h. The SWCNT layer was then prepared by depositing a droplet of 2 μL of the SWCNT solution three times (total 120 ng of SWCNT deposited) onto the screen-printed interdigitated array (IDA) microelectrodes (gold electrodes on a corundum ceramic base, CC1.W1, batch # 12C12, BVT technologies, Brno, CZ) to have 1~10 kΩ range of the baseline resistance. The interdigitated array microelectrodes consist of 3 pairs of gold electrodes with 150 μm spacing between electrodes, 150 μm electrode width, and 2000 μm electrode length. The functionalized cellulose acetate was then deposited by adding three droplets of 2 μL of the solution of 1 mg/mL (acetone) on top of the SWCNT layer (total 6 μg deposited). The sensor of the pristine SWCNT was also prepared by depositing a droplet of 2 μL of the freshly dispersed SWCNT solution (20 μg/mL in THF) three times. All sensors were dried for 12 h at ambient condition before use. The photographs and SEM images of devices were given in Figure 3. Less of the SWCNTs network was observed after overcoating the functionalized cellulose acetate layer on top of the SWCNTs layer (Figure 3d), as compared to bare SWCNTs network on the IDA (Figure 3c).

We estimated the thickness of the SWCNT and cellulose layer based on the mass deposited on the sensor as follows:

(2) $$t=\frac{m}{d\times A}$$

where *t*, *m*, *d*, and *A* are thickness, mass, density, and area, respectively. We used 1.8 g/cm^3^ as the average density of SWCNTs is provided by the chemical vendor (Aldrich). The estimated density of functionalized cellulose was taken from the density of cellulose acetate of 1.31 g/cm^3^, which is also provided by the vendor (Eastman). The area of the sensor (*A*) was 0.04 cm^2^. By these calculations the effective average SWCNT layer thickness is 17 nm thick and it is important to note that this film is not expected to be continuous, but an interconnected random network of carbon nanotubes. The cellulose layer thickness is calculated to be 1200 nm by this method.

### 2.6. Sensing Measurements

The sensing properties were measured by monitoring the conductivity change of sensors upon exposure to analytes at 23 °C. The sensors deposited on IDAs were mounted on 2 × 30 pin edge connector and encased within a custom-built Teflon chamber with an inlet, an outlet, and an internal channel with volume of 7.38 cm^3^ for gas flow. The analyte vapors of varying concentrations were generated using FlexStream^TM^ FlexBase Module with precise temperature (±0.01 °C) and gas flow rate control (±1.5% of the reading). The vapor of varying concentrations was generated by mixing the saturated vapor with dry nitrogen gas and was delivered to the sensor chamber. The conductivity measurement was carried out by measuring the current at 0.05 V bias voltage using a PalmSense EmStat-MUX equipped with a 16 channel multiplexer (Palm Instruments BV, The Netherlands), and the baseline resistances of sensors were in the range of 1 kΩ to 10 kΩ. The sensors were tested by measuring the changes in conductance after several cycles of exposure to the analyte vapor for 1 min, and they were recovered by purging nitrogen after exposure to the analyte. The normalized conductance change [−Δ*G*/*G_o_* (%)] was calculated from the current reading in response to an analyte using the following equation:

(3) $$-\Delta G/G_{o}(\%)=\frac{\left(I_{o}-I\right)}{I_{o}}\times 100$$

where *I_o_* is the baseline current and *I* is the current of a sensor after exposure to an analyte. The sensor experiences a linear baseline drift of ≈0.04% after each exposure and for a given sensor the shift was reproducible over many exposures. The data in the figures has a simple linear baseline correction applied.

## 3. Results and Discussion

**Figure 4:**
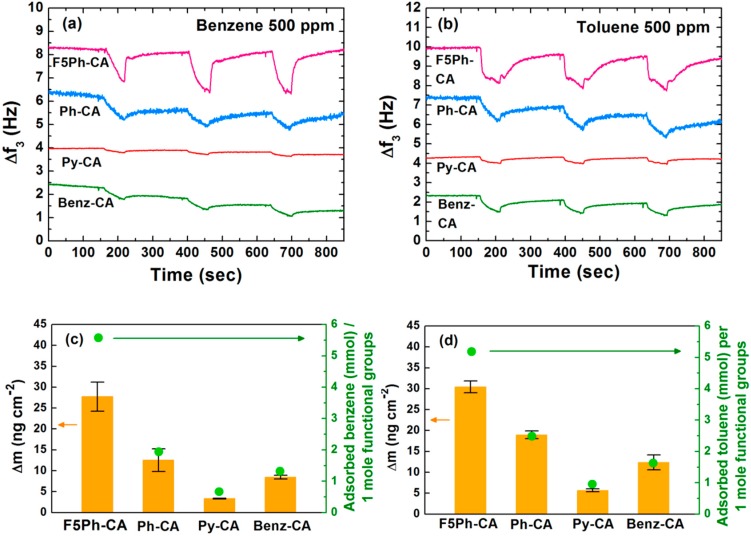
Frequency changes (Δ*f*_3_, 3rd overtone) and mass uptake (Δ*m*) of the four functionalized cellulose acetates of F5Ph-CA, Ph-CA, Py-CA, and Benz-CA when exposed to 500 ppm of benzene and toluene vapors: (**a**,**b**) frequency changes upon exposure to benzene and toluene, respectively; (**c**,**d**) mass uptakes upon exposure to benzene and toluene, respectively.

QCM experiments were performed to test the binding ability of the functionalized cellulose acetate towards the target vapor. QCM is a gravimetric sensor which can be used to investigate the sorption of an analyte into a polymer film, and the response is only dependent on the analyte property that influences sorption [24]. Mass uptake of the functionalized cellulose acetate film was measured by monitoring the frequency change of the film upon exposure to the target vapor. The films were tested towards 500 ppm of benzene (0.47% of the saturated vapor) and toluene (1.3% of the saturated vapor) with dry nitrogen carrier gas. Figure 4 shows the frequency and mass density changes of the four functionalized cellulose acetate films including F5Ph-CA, Ph-CA, Py-CA, and Benz-CA when exposed to benzene and toluene vapors. As shown in Figure 4a,b, the frequencies (the 3rd overtone, *f_3_*) of the four functionalized cellulose acetate films decreased upon exposure to both benzene and toluene vapors due to the adsorbed vapor molecules on the films, and the responses were reversible in all cases.

The measured frequency change (Δ*f*) was converted to the mass density change (Δ*m*) using Sauerbrey’s equation. The adsorbed amount (mmol) of an analyte per 1 mole of functional groups was then calculated from Δ*m* and degrees of substitution of functional groups and was summarized in Figure 4c,d. The QCM results reveal that F5Ph-CA films have superior binding ability towards benzene and toluene vapors, compared to the other films. Specifically the mass density change of the F5Ph-CA film upon exposure to benzene vapor of 500 ppm was more than two times higher than those of the other films. The abilities of the four functionalized cellulose acetates to adsorb toluene vapor had similar trends to those in response to benzene vapor. Direct comparison of the mass uptakes between the F5Ph-CA (5.576 mmol of adsorbed benzene per 1 mole of F5Ph selectors) and Ph-CA (1.937 mmol of adsorbed benzene per 1 mole of Ph selectors) films proved our hypothesis in that the F5Ph selectors with positive electrostatic potential on their π-surface had stronger interactions with benzene and toluene possessing negative quadrupole moments below and above the aromatic ring, which is consistent with the previous literature on the strong interaction between arene and perfluoroarene [25]. The two products containing triazole rings, Py-CA and Benz-CA, showed less absorption efficiency towards benzene and toluene vapors.

**Figure 5:**
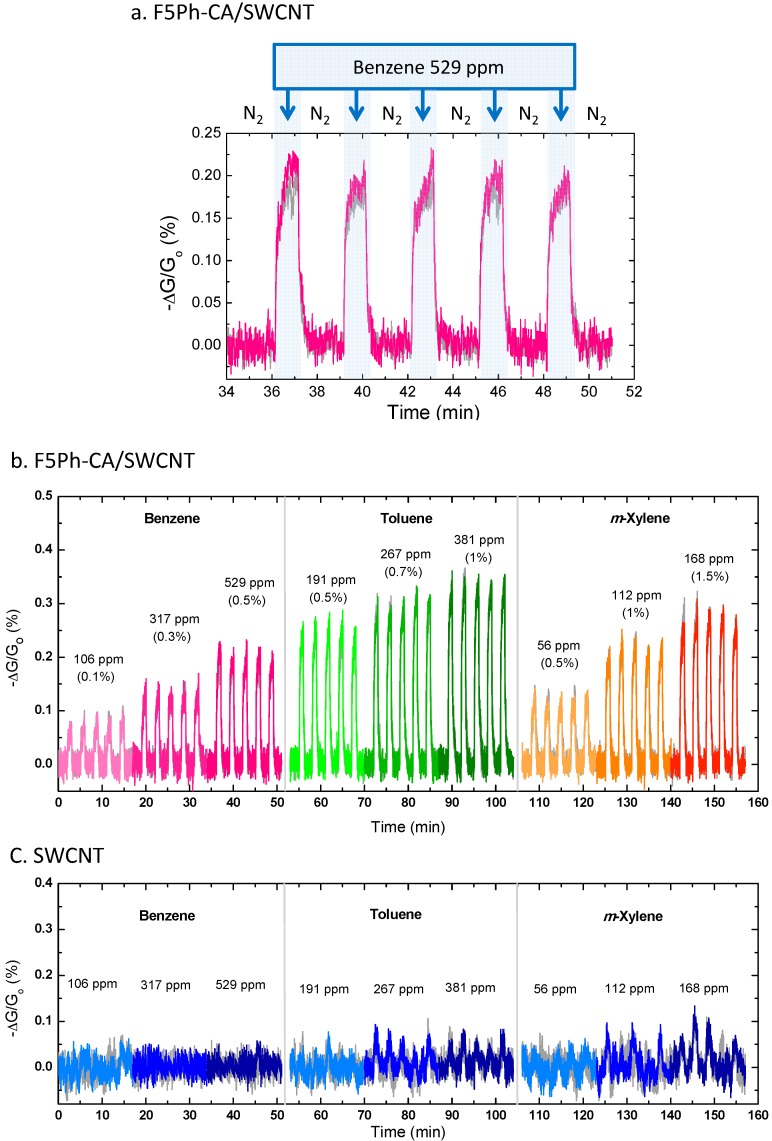
(**a**) Normalized conductance changes [−Δ*G*/*G_o_* (%)] of the integrated F5Ph-CA concentrator/SWCNT system (F5Ph-CA/SWCNT) when exposed to benzene of 529 ppm, (**b**) normalized conductance changes of F5Ph-CA/SWCNT when exposed to benzene, toluene, and *m*-xylene vapors of varying concentrations compared to the pristine SWCNT sensor (**c**). The responses from a set of duplicate sensors were consistent, as shown by the grey curves representing the sensing response of the second sensor.

The integrated polymer concentrator/sensor system was fabricated by dropcoating SWCNTs freshly dispersed by sonication onto IDA followed by dropcoating functionalized cellulose acetate on top of the SWCNT sensing layer. The F5Ph-CA with DS_F5Ph_ of 0.80 was chosen as a polymer concentrator since it showed superior adsorbing abilities towards benzene and toluene vapors from the QCM results. The array consisting of the pristine SWCNTs and the integrated F5Ph-CA polymer concentrator/SWCNT system (F5Ph-CA/SWCNT) was tested by simultaneously exposing it to an analyte vapor and measuring the interelectrode current at 0.05 V bias voltage. Figure 5 shows the normalized conductance changes [−Δ*G*/*G_o_* (%)] of the pristine SWCNTs and F5Ph-CA/SWCNT sensors towards benzene, toluene, and *m*-xylene vapors of varying concentrations. The normalized conductance changes of both sensors were averaged from two devices of each sensor. As shown in Figure 5, the conductance of the F5Ph-CA/SWCNT sensor decreased within a few seconds when exposed to benzene, toluene, and *m*-xylene vapors and the responses of the sensing system were reproducible. The average time at which the response [−Δ*G*/*G_o_* (%)] reached 90% of the equilibrium state was 7 s (±24%), and the average time necessary for complete recovery of the response was 20 s (±28%). The F5Ph-CA/SWCNT sensor was able to detect benzene vapor down to 106 ppm (0.1% of the saturated benzene vapor), whereas the pristine SWCNTs did not show a detectable response at the low concentrations we tested. Considering the combined data from Figure 4 and Figure 5 the F5Ph-CA layer in the F5Ph-CA/SWCNT sensing system serves as the concentrating layer and adsorbs and concentrates the analyte vapor from the gas phase. Subsequently, the sorption of the analyte into the layer is expected to perturb the electronic and dipolar interactions at the polymer-SWCNT interface, which could result in increased intra-SWCNT resistivity by carrier scattering/pinning mechanisms. It is also expected that sorption will increase the inter-SWCNT resistance as the physical presence of analyte in the films can cause expansion and increase the distance required for tunneling conductance between carbon nanotubes. The latter mechanism is similar to those observed for chemiresistive sensors based upon carbon/polymer composites [26] and nanoparticles coated with self-assembled monolayers [27,28,29]. We also conducted control experiments with sensor devices prepared from dropcasting films of the premixed SWCNTs and F5Ph-CA (2% weight percent of SWCNTs in F5Ph-CA) solution. This sensor like the bare SWCNTs did not show any response to benzene vapor of 500 ppm and displayed more baseline noise than the integrated cellulose overcoated system on SWCNTs (Figure S7). Hence, placing the F5Ph-CA layer on top of a preassembled SWCNT network provides a superior sensor system. The detection limits of the F5Ph-CA/SWCNT sensor towards benzene, toluene, and *m*-xylene vapors were calculated to be 55 ppm, 19 ppm, and 14 ppm, respectively, from the Figure 5 when the signal equaled three times the noise level (Figure S8). The detection limit of the system to benzene was higher than the Occupational Safety and Health Administration (OSHA) permissible exposure limit (PEL) of 1 ppm [30,31,32] as eight-hour time-weighted average concentration, but detection limits for toluene and *m*-xylene vapors are substantially under the PELs of 200 ppm and 100 ppm, respectively.

**Figure 6:**
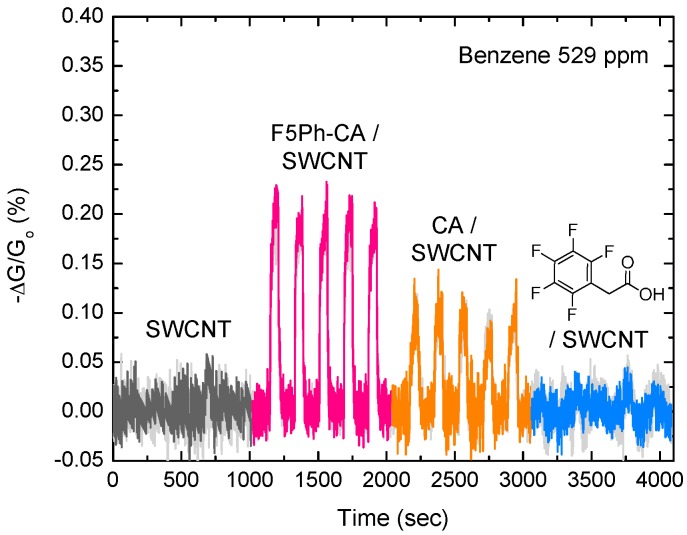
Normalized conductance changes of SWCNT, F5Ph-CA/SWCNT, CA/SWCNT, and 2,3,4,5,6-pentafluorophenylacetic acid/SWCNT) systems toward 529 ppm of benzene vapor.

The CA/SWCNT (CA with DS_Ac_ ~ 2.44) and (2,3,4,5,6-pentafluorophenylacetic acid)/SWCNT were also tested with 529 ppm (0.5% of the saturated vapor) of benzene vapor to demonstrate the high efficiency of the F5Ph-CA concentrator. Figure 6 shows that the integrated F5Ph-CA/SWCNT sensing system has a superior ability to detect benzene vapor, compared to the other systems. Depositing 2,3,4,5,6-pentafluorophenylacetic acid of a similar loading to the cellulose acetate system on SWCNT system did not show any response to benzene vapor, proving the critical role of the functionalized cellulose acetates. The CA/SWCNT sensing system also showed the response to benzene vapor of 529 ppm, but the response was half of that of the F5Ph-CA/SWCNT system. Better response of the F5Ph-CA/SWCNT system, compared to CA/SWCNT system, might be due to the better interfacial compatibility between the F5Ph functional groups of cellulose acetate and the sidewall of SWCNTs induced from the electrostatic interaction, which can be affected by the sorption-induced interface change. Therefore, the F5Ph selector is effective at adsorbing and concentrating benzene molecules.

**Figure 7:**
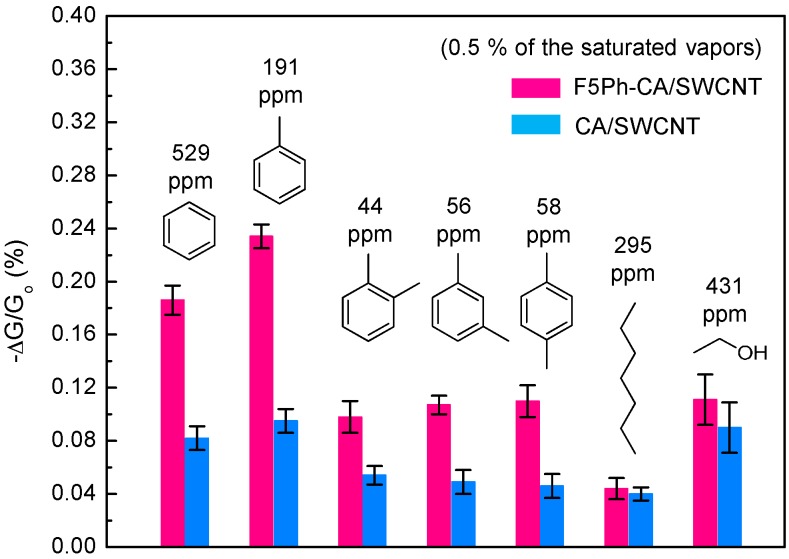
Selectivity chart displaying the responses of the F5Ph-CA/SWCNT and CA/SWCNT systems towards BTX vapors and *n*-heptane and ethanol as interferents. The concentrations of the analytes were 0.5% of the saturated vapors.

The selectivity of the F5Ph-CA/SWCNT sensor was studied by testing the system in response to benzene, toluene, *o*-xylene, *m*-xylene, *p*-xylene, *n*-heptane, and ethanol of 0.5% of the saturated vapors. *N*-heptane and ethanol are commonly found in petrochemicals and hence were tested as interferents. Figure 7 displays the normalized conductance change of the array of the F5Ph-CA/SWCNT and CA/SWCNT sensing systems. The integrated F5Ph-CA/SWCNT system had differential responses to benzene, toluene, and xylenes although it had equivalent responses to the different isomers of xylenes. The responses of the F5Ph-CA/SWCNT to xylenes and ethanol vapors were shown to be similar. However, xylenes and ethanol can be differentiated by having the CA/SWCNT sensor as the part of the array since the responses of the CA/SWCNT to xylenes were 50% of those of the F5Ph-CA/SWCNT, whereas the response of the CA/SWCNT to ethanol was similar to that of the F5Ph-CA/SWCNT. The F5Ph-CA/SWCNT sensor showed the least sensitivity towards *n*-hexane, which was 24% and 19% of the responses to benzene and toluene vapors, respectively. The selectivity tests demonstrate that the F5Ph-CA/SWCNT coupled with the CA/SWCNT sensing system contributes to differentiate benzene, toluene, and xylenes from *n*-heptane and ethanol interferents.

We also studied the influence of humidity on the sensor performance using compressed air with different humidity levels as a carrier gas. The normalized conductivity change (−Δ*G*/*G_o_* (%)) of the F5Ph-CA/SWCNT system was measured when exposed to benzene of varying concentrations in the presence of air with humidity levels of 8.5% and 66% (Figure S9). The F5Ph-CA/SWCNT system detected benzene vapor of 529 ppm in the air with humidity of 8.5%, which was not as strongly as in dry nitrogen as a carrier gas. The responses of the sensors upon exposure to benzene vapor of 529 ppm in the presence of air with humidity level of 8.5% decreased almost 98%, compared to the responses toward benzene vapor of 529 ppm in dry nitrogen. Under high humidity condition (66%), no response was observed at any benzene concentration. It might be due to the unreacted hydroxyl groups of functionalized cellulose acetates which decreased in sensitivity of the system under high humidity condition. We are currently making efforts to mitigate the humidity effects in ongoing materials designs so that this concept can find application in uncontrolled environmental conditions.

## 4. Conclusions

In summary, we have demonstrated a new design for a BTX sensor based on the integrated polymer concentrator/SWCNT sensing system where a functionalized cellulose concentrating layer was deposited directly on top of the SWCNT sensing layer, allowing concentrating and sensing simultaneously within a few seconds. The cellulose acetates functionalized with selectors for target analytes were successfully demonstrated as a concentrator in this study. The functionalized cellulose acetate concentrating layer selectively adsorbed the target analyte and delivered the concentrated analyte to the SWCNT sensing layer, which allowed the system to detect tens of ppm of BTX vapors. The detection limits towards toluene and xylenes are significantly lower than the OSHA permissible exposure limit. Interdigitated array microelectrodes with smaller gaps represent an approach to produce lower detection limits for BTX gases. The high sensitivity, selectivity, and fast response are significant benefits of the integrated polymer concentrator/sensing system. The decreased sensitivity under high humidity conditions, however, remains one of the challenges with this system for on-site field monitoring applications. The further investigation on the system design is currently under way to mitigate the humidity effect on the sensitivity.

## Supplementary Files

Supplementary File 1
