# Supplementary material for: Integrated Gas Sensing System of SWCNT and Cellulose Polymer Concentrator for Benzene, Toluene, and Xylenes
Source: Sensors (Basel). 2016 Feb 2;16(2):183. doi: 10.3390/s16020183 (PMC4801560; doi:10.3390/s16020183)
Supplement: Supplementary File 1 [file sensors-16-00183-s001.pdf]

Supplementary Materials: Integrated Gas Sensing System of SWCNT and Cellulose Polymer Concentrator for Benzene, Toluene, and Xylenes

Jisun Im, Elizabeth S. Sterner and Timothy M. Swager

Experimental Section

- Syntheses of Selectors……………………………………………………………..........S2
  - A. Propargyl Pyrenebutyl Ether………………………………………………….......S2
  - B. 2,3,4,5,6-Pentafluorophenylacetyl Chloride (F5Ph-Cl)……………………….............S5
  - C. Phenylacetyl Chloride (Ph-Cl)………………………………………………………..S5
- Syntheses of Functionalized Cellulose Acetates……………………………………........S4
  - A. 6-Deoxy-6-Azido Cellulose Acetate (CA-N_3)………………………………………..S4
  - B. Pyrene-Functionalized Cellulose Acetate (Py-CA)……………………………….........S5
  - C. Benzoate-Functionalized Cellulose Acetate (Benz-CA)………………………………S7
  - D. 2,3,4,5,6-Pentafluorophenylacetyl-Functionalized Cellulose Acetate (F5Ph-CA)......S8
  - E. Phenylacetyl-Functionalized Cellulose Acetate (Ph-CA)……………………………...S9
- Calculation of the Detection Limit……………………………………………….............S11
- Influence of Humidity on Sensor Performance……………………………………………...S12
- References…………………………………………………………………………………..S14

Figures

Figure S1. ^{1}H NMR (top) and ^{13}C NMR (bottom) spectra of propargyl pyrenebutyl ether….....S2
Figure S2. ^{1}H NMR (top) and ^{13}C NMR (bottom) spectra of CA-N_3……………………………S4
Figure S3. ^{1}H NMR (top) and ^{13}C NMR (bottom) spectra of Py-CA………………………………S6
Figure S4. ^{1}H NMR (top) and ^{13}C NMR (bottom) spectra of Benz-CA…………………............S7

Figure S5. ^{1}H NMR (top) and ^{13}C NMR (bottom) spectra of F5Ph-CA……………………………S9

Figure S6. ^{1}H NMR (top) and ^{13}C NMR (bottom) spectra of Ph-CA………………………………S10

Figure S7. Normalized conductance changes [$-\Delta G/G_o$ (%)] of the device prepared from (**a**) the premixed F5Ph-CA polymer and SWCNTs and (**b**) the integrated F5Ph-CA concentrator/SWCNT system (F5Ph-CA/SWCNT) when exposed to benzene vapor……..………………………………S11
Figure S8. Normalized conductance changes of the F5Ph-CA/SWCNT sensor as a function of concentration of (**a**) benzene, (**b**) toluene, and (**c**) *m*-xylene vapors showing the limit of detection (LOD) where the signal equals three times the noise………………………………………………S12
Figure S9. Normalized conductance changes [$-\Delta G/G_o$ (%)] of the integrated F5Ph-CA concentrator/SWCNT system (F5Ph-CA/SWCNT) when exposed to benzene in the presence of humid air: (**a**) benzene of 529 ppm with a humidity level of 8.5%, (**b**) benzene of 529 ppm with a humidity level of 66%, (**c**) benzene of 400 ppm with a humidity level of 8.5%, (**d**) benzene of 400 ppm with a humidity level of 66%, (**e**) benzene of 250 ppm with a humidity level of 8.5%, and (**f**) benzene of 250 ppm with a humidity level of 66%. In all figures, the bottom trace (green) was the response of the pristine SWCNT sensor, while the responses of a triplet of the F5Ph-CA/SWCNT were shown to be blue, red, and aqua……………………………………………………………………………..S13

Experimental Section

Syntheses of Selectors

A. Propargyl Pyrenebutyl Ether

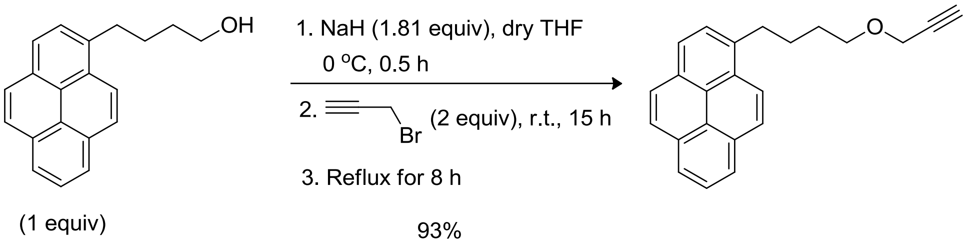

The reaction was conducted by the previously reported procedure [1]. Sodium hydride (NaH, 60 wt% in oil, 0.633 g, 26.39 mmol) was added to a solution of 1-pyrenebutanol (4 g, 14.58 mmol) in dry THF (45 mL) at 0 °C under argon atmosphere. The mixture was stirred at 0 °C for 30 min, and propargyl bromide (80 wt% in toluene, 3.469 g, 29.16 mmol) was added. The solution was stirred at 0 °C in the dark for 30 min and allowed to warm up to room temperature. After stirring for 15 h at room temperature, the reaction was refluxed for 8 h. Ethyl acetate (20 mL) and distilled water (20 mL) were added to the solution, and the aqueous phase was extracted twice with 20 mL of ethyl acetate. The combined organic phase was washed with 20 mL of brine and dried over $MgSO_4$. The solvent was removed under vacuum, and the crude product was purified by column chromatography using toluene as an eluent to get the product (4.268 g, 93%). ^{1}H NMR (500 MHz, $CDCl_3$, δ): 1.82 (m, 2H), 1.96 (m, 2H), 2.46 (t, J = 2.4 Hz, 1H), 3.38 (t, J = 7.7 Hz, 2H), 3.60 (t, J = 6.4 Hz, 2H), 4.17 (d, J = 2.4 Hz, 2H), 7.87~8.32 (m, 9H); ^{13}C NMR (300 MHz, $CDCl_3$, δ): 28.46, 29.66, 33.33, 58.19, 70.05, 74.31, 80.12, 123.55, 124.75, 124.87, 124.91, 125.12, 125.16, 125.86, 126.64, 127.27, 127.61, 136.81; HRMS (DART) *m*/*z*: $[M + H]^+$ calcd. for $C_{23}H_{20}O$, 313.1587; found, 313.1571.

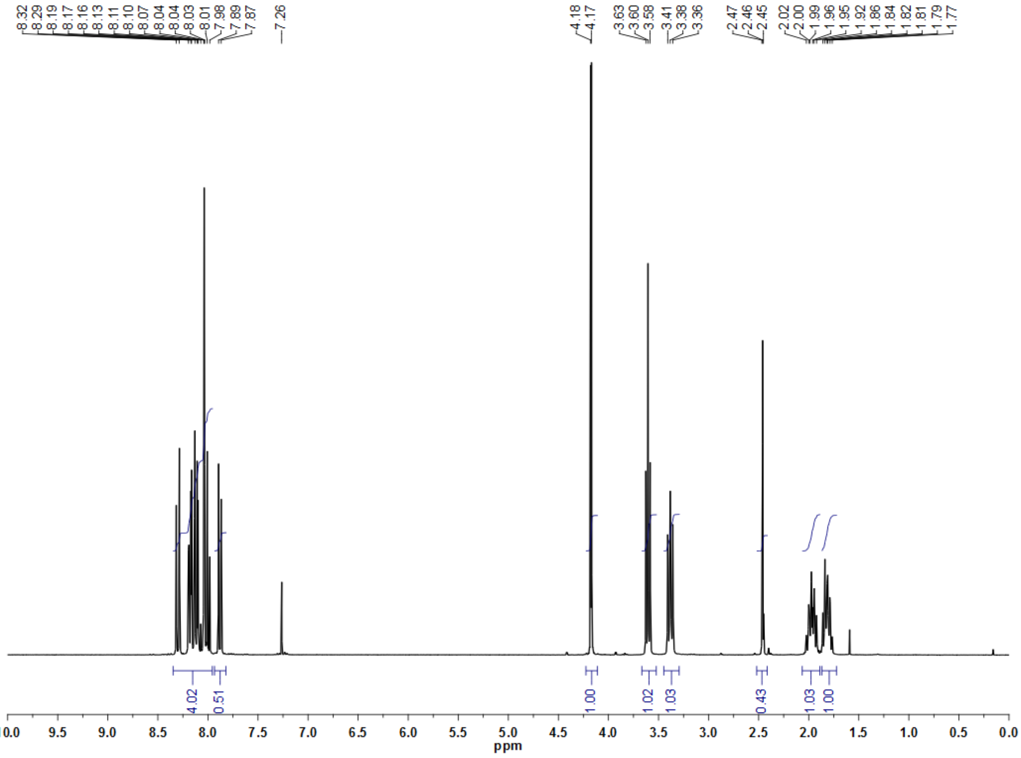

Figure S1. *Cont.*

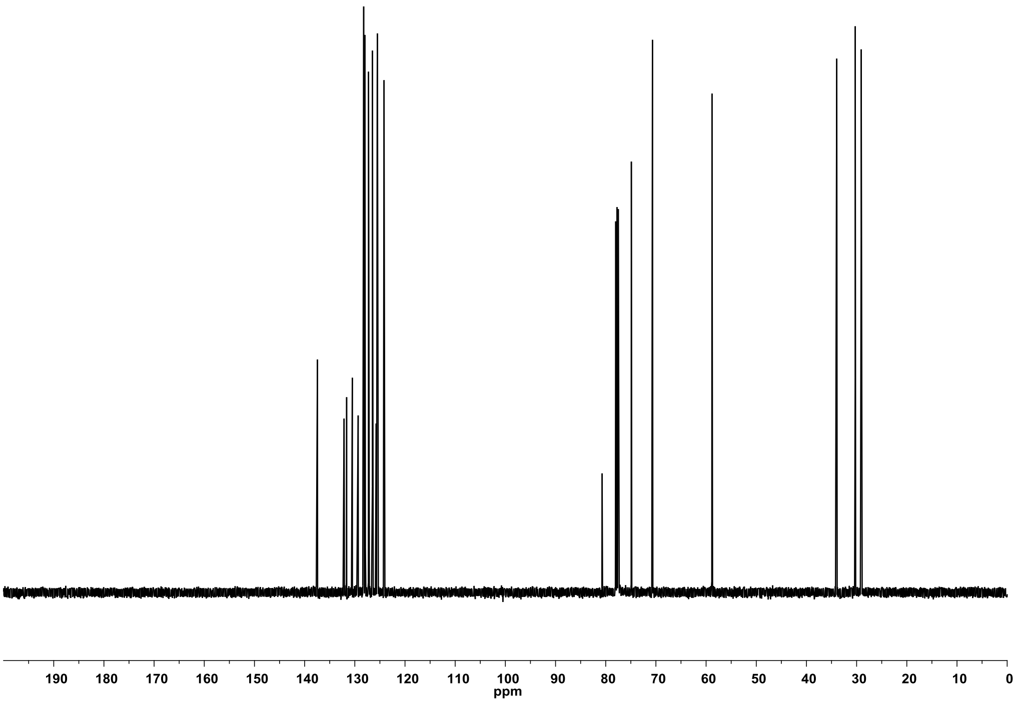

Figure S1. ^{1}H NMR (**top**) and ^{13}C NMR (**bottom**) spectra of propargyl pyrenebutyl ether.

B. 2,3,4,5,6-Pentafluorophenylacetyl Chloride (F5Ph-Cl)

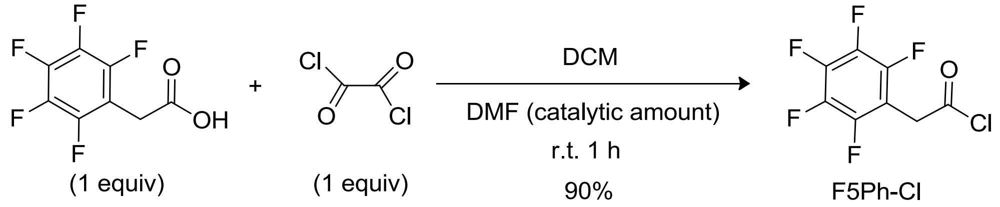

The synthesis was carried out by following the previously reported method [2]. To a solution of 2,3,4,5,6-pentafluorophenylacetic acid (2 g, 8.84 mmol) in 100 mL of CH_2Cl_2 in an ice bath was added dropwise oxalyl chloride (1.122 g, 8.84 mmol). Anhydrous DMF (catalytic amount) was then added. The solution was allowed to warm to room temperature during 2 h, and CH_2Cl_2 was removed under vacuum. The product was kept in a refrigerator and used within 2 days. ^{1}H NMR (500 MHz, $CDCl_3$, δ): 4.26 (s, 2H); ^{19}F NMR (300 MHz, $CDCl_3$, δ): −141.93, −152.99, −161.49.

C. Phenylacetyl Chloride (Ph-Cl)

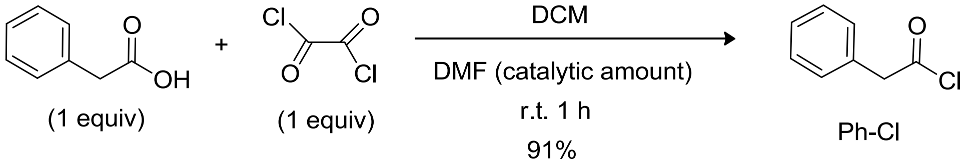

Phenylacetyl chloride was prepared by the same procedure for F5Ph-Cl. ^{1}H NMR (500 MHz, $CDCl_3$, δ): 4.15 (s, 2H), 7.21–7.40 (m, 5H).

Syntheses of Functionalized Cellulose Acetates

A. 6-Deoxy-6-Azido Cellulose Acetate (CA-N_3)

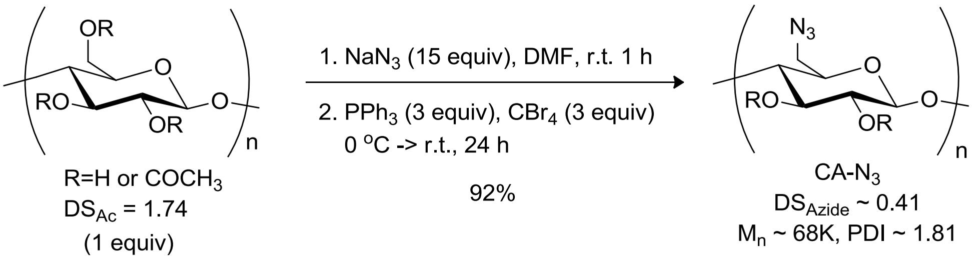

Cellulose acetate (CA) with the acetyl content of 31.9 wt% (DS_{Ac} ~ 1.74) was employed as a starting material. It's soluble in DMSO and DMF, and 51 % of the primary hydroxyl groups at C6 position (estimated from ^{13}C NMR spectrum) can be utilized for further functionalization while keeping acetate functional groups. Typically azidation is carried out via two step reaction: bromination or tosylation of hydroxyl groups and then azidation using sodium azide. However, bromination or tosylation and displacement with azide proved ineffective with cellulose acetate as a starting material. Here, one pot azidation reaction was carried out to produce 6-deoxy-6-azido cellulose acetate (CA-N_3) with 92% yield by modifying the previously reported procedure [3]. Briefly, cellulose acetate (2 g, 8.13 mmol) was premixed with excess sodium azide (NaN_3, 8.125 g, 125 mmol) in 50 mL of DMF at room temperature for 1 h. In this step, heating to 100 °C helped to dissolve cellulose acetate in DMF faster. However, it resulted in lower yield (84%). Triphenylphosphine (PPh_3, 6.400 g, 24.4 mmol) was added to the solution at 0 °C, and carbon tetrabromide (CBr_4, 8.092 g, 24.4 mmol) in 10 mL of DMF was then added dropwise to the solution. The mixture was allowed to warm to room temperature and stirred for 24 h. The degree of substitution of azide was calculated to be 0.41 from elemental analysis. The polymer was precipitated in 700 mL of methanol while stirring. The filtered polymer was further washed with 500 mL of methanol and dried under vacuum at 40 °C for 4 h. The degree of substitution of azide (DS_{Azide}) was 0.41 from elemental analysis, and CA-N_3 was soluble in acetone, THF, DMF, and DMSO. M_n: 68 KDa, M_w: 123 KDa, PDI: 1.81; Elemental analysis: C 45.87, H 4.86, N 6.94.

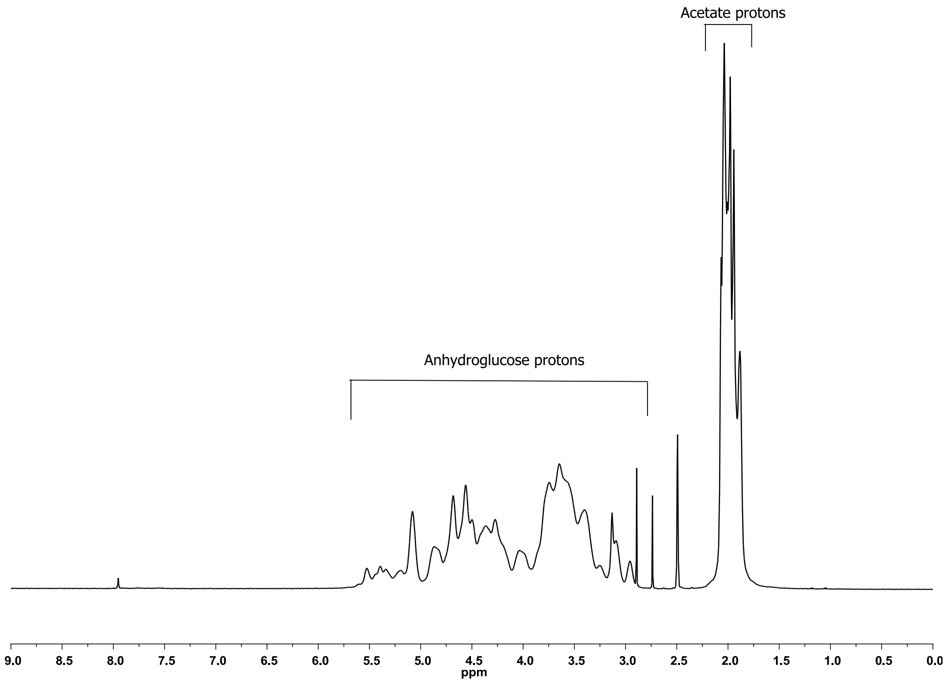

Figure S2. *Cont.*

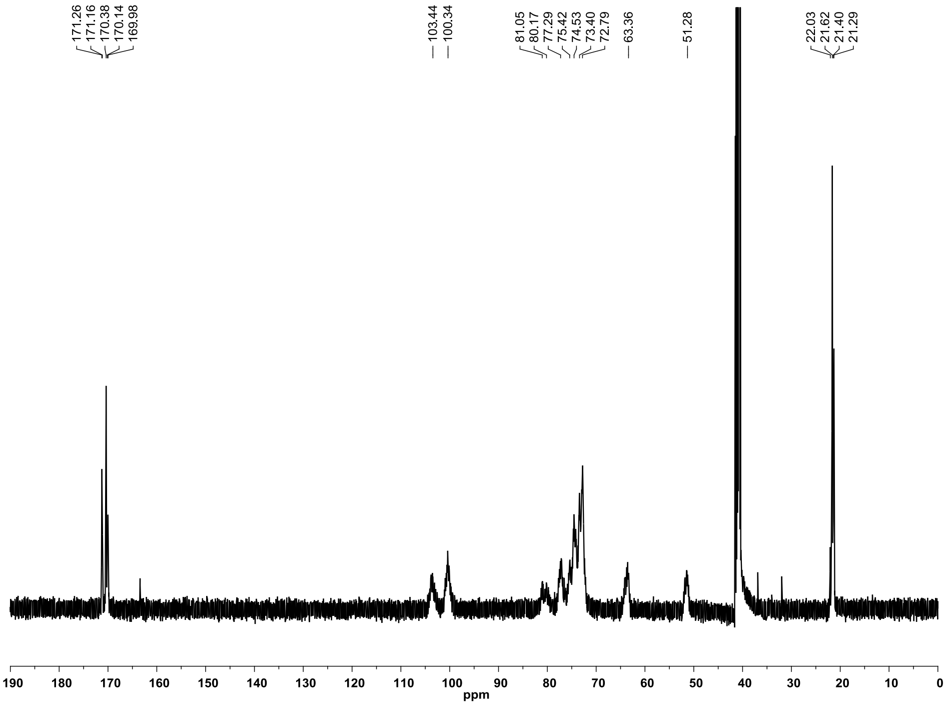

Figure S2. ^{1}H NMR (**top**) and ^{13}C NMR (**bottom**) spectra of CA-N_3.

B. Pyrene-Functionalized Cellulose Acetate (Py-CA)

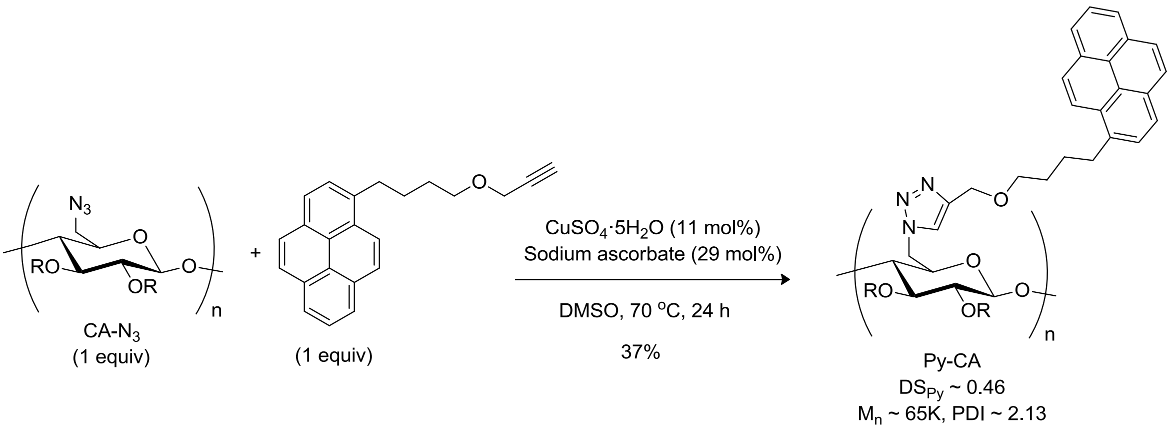

6-Deoxy-6-azide cellulose acetate (0.1 g, ~0.435 mmol) was dissolved in 10 mL of DMSO, and $CuSO_4 \cdot H_2O$ (12.4 mg, 11 mol%) in 0.5 mL of distilled water and sodium ascorbate (26.4 mg, 29 mol%) in 0.5 mL of distilled water were added to the solution. Propargyl pyrenebutyl ether (0.136 g, 0.435 mmol) in 3 mL of DMSO was added, and the solution was heated to 70 °C while stirring for 24 h under dark. The polymer was precipitated in 200 mL of methanol while stirring. It took almost 2 h for the polymer to be completely precipitated in methanol. The filtered polymer was further washed with 100 mL of H_2O and 100 mL of methanol and dried under vacuum at room temperature. The degree of substitution of pyrene selector (DS_{Py}) was 0.46 from elemental analysis, and Py-CA was soluble in DMSO and THF. M_n: 65 KDa, M_w: 138 KDa, PDI: 2.13; Elemental analysis: C 59.36, H 5.11, N 4.82, Cu 0.63.

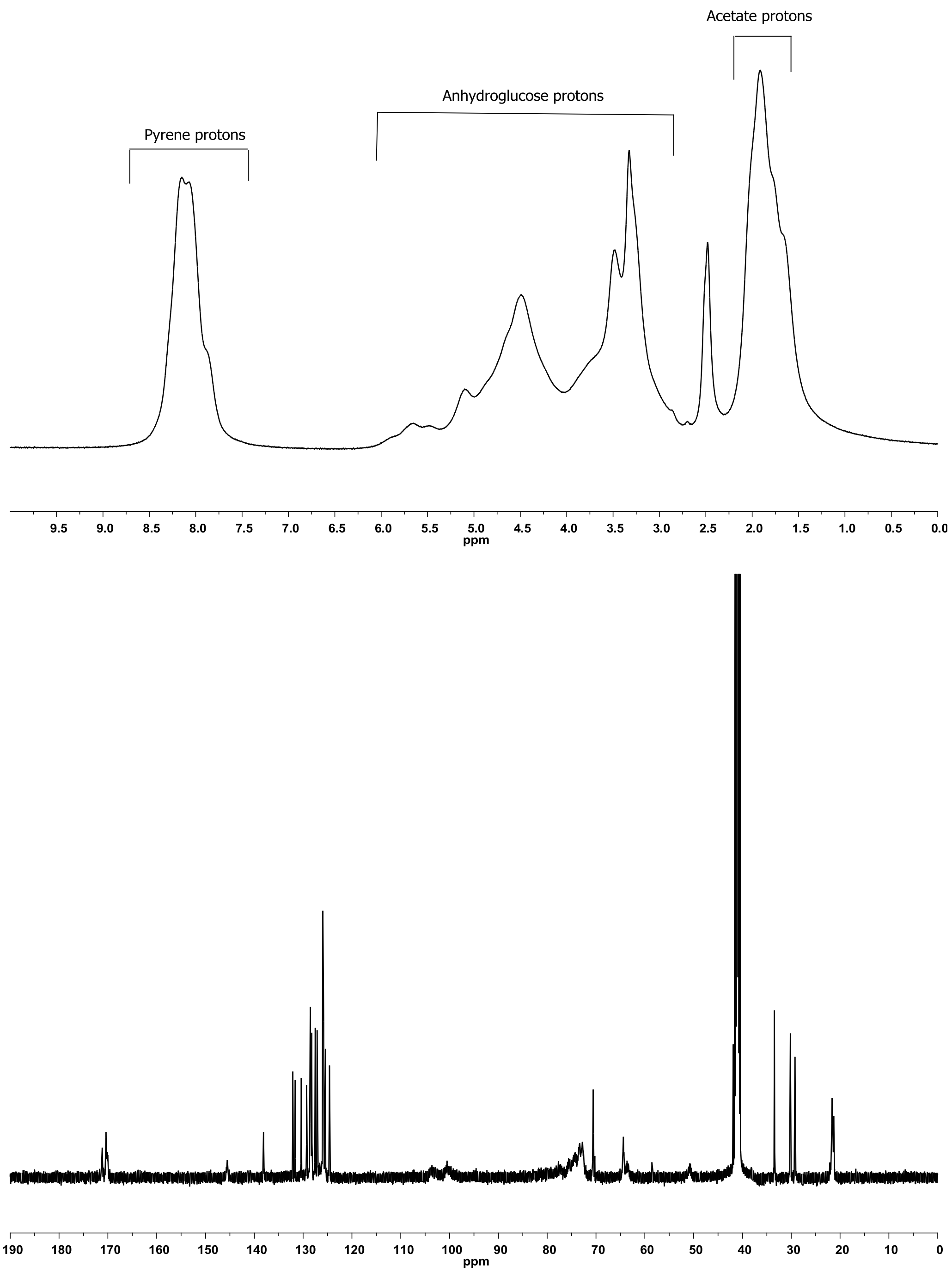

Figure S3. ^{1}H NMR (**top**) and ^{13}C NMR (**bottom**) spectra of Py-CA.

C. Benzoate-Functionalized Cellulose Acetate (Benz-CA)

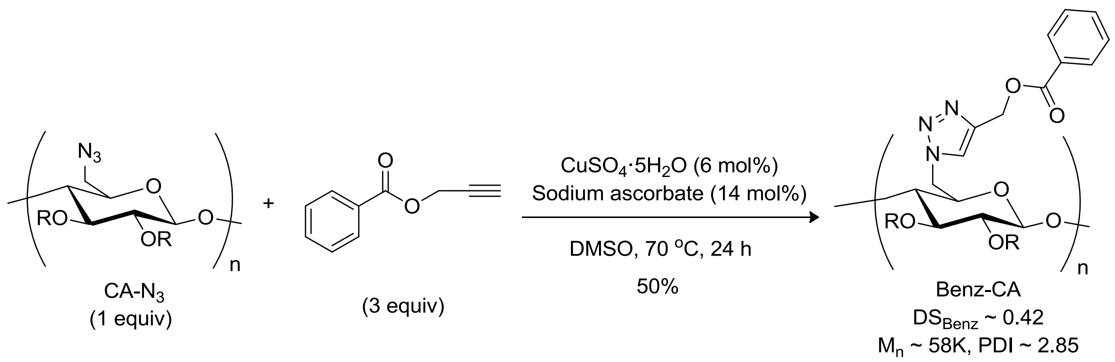

The synthetic procedure is the same as that for Py-CA except for the following condition: $CuSO_4 \cdot H_2O$ (6 mol%), sodium ascorbate (14 mol%), and propargyl benzoate (3 equiv). The degree of substitution of benzoate selector (DS_{Benz}) was 0.42 from elemental analysis, and Benz-CA was soluble in DMSO and THF. M_n: 58 KDa, M_w: 166 KDa, PDI: 2.85; Elemental analysis: C 50.37, H 4.72, N 5.69, Cu 1.35.

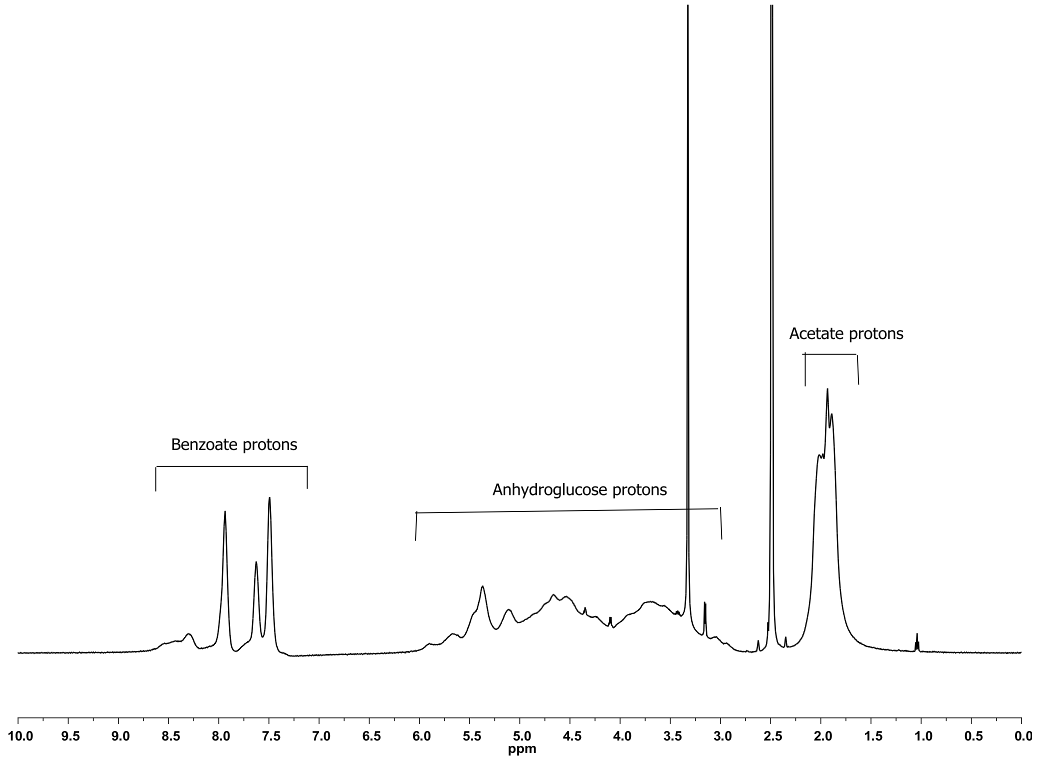

Figure S4. *Cont.*

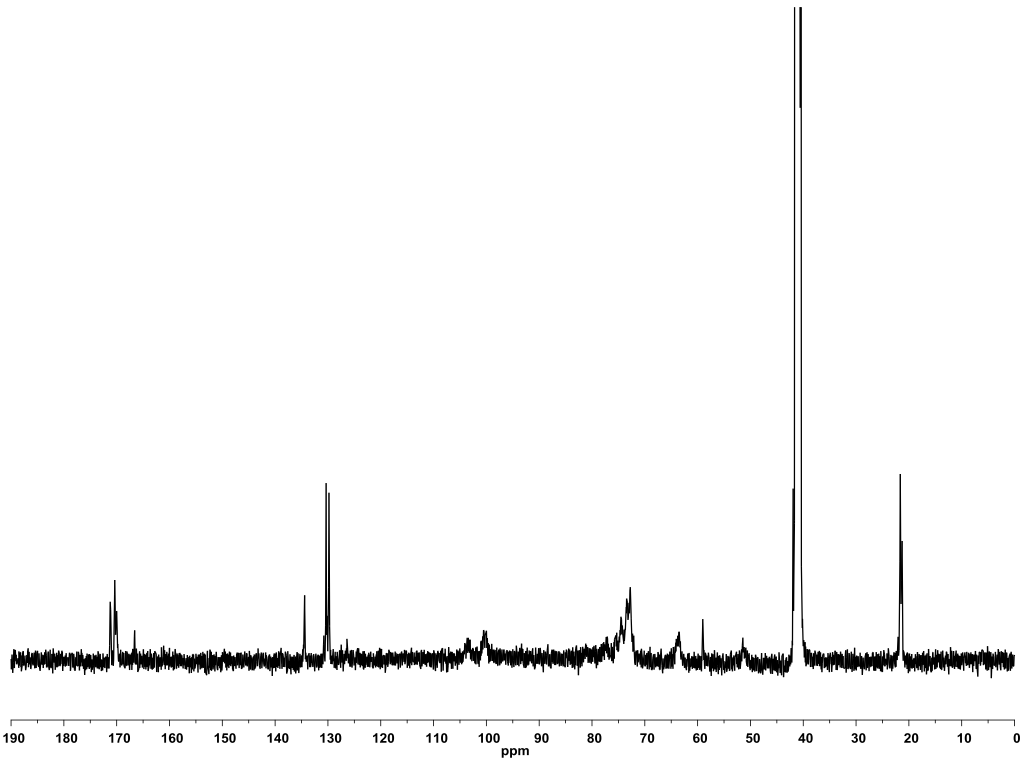

Figure S4. ^{1}H NMR (**top**) and ^{13}C NMR (**bottom**) spectra of Benz-CA.

D. 2,3,4,5,6-Pentafluorophenylacetyl-Functionalized Cellulose Acetate (F5Ph-CA)

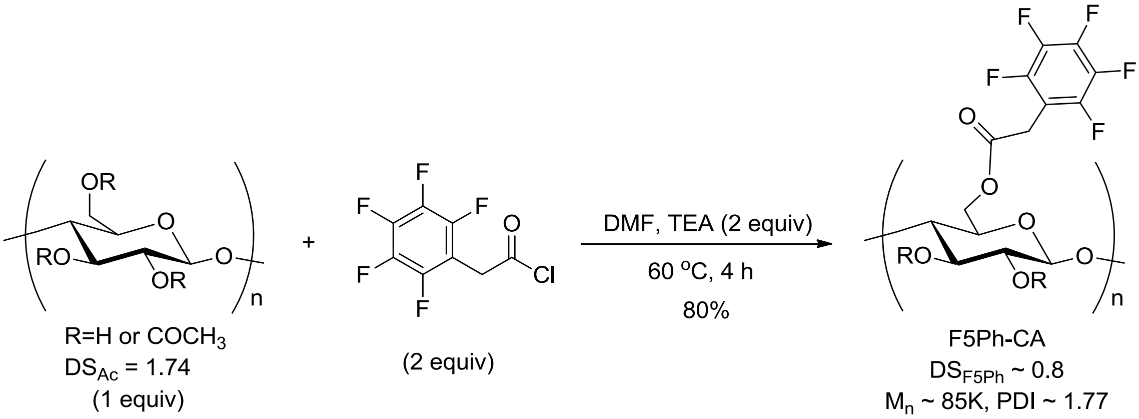

F5Ph-Cl (1.780 g, 7.317 mmol) was added dropwise to a solution of CA (0.9 g, 3.658 mmol) in 20 mL of DMF at 60 °C. Triethylamine (0.74 g, 7.317 mmol) was then added as a catalyst, and the mixture was stirred for 4 h at 60 °C. The polymer was precipitated in 400 mL of methanol. The filtered product was further washed with 200 mL of methanol and dried under vacuum. The degree of substitution of F5Ph selector (DS_{F5Ph}) was 0.80 from elemental analysis, and F5Ph-CA was soluble in acetone, THF, DMSO, and DMF. M_n: 85 KDa, M_w: 150 KDa, PDI: 1.77; Elemental analysis: C 47.46, H 2.62, N 0.28, F 18.98.

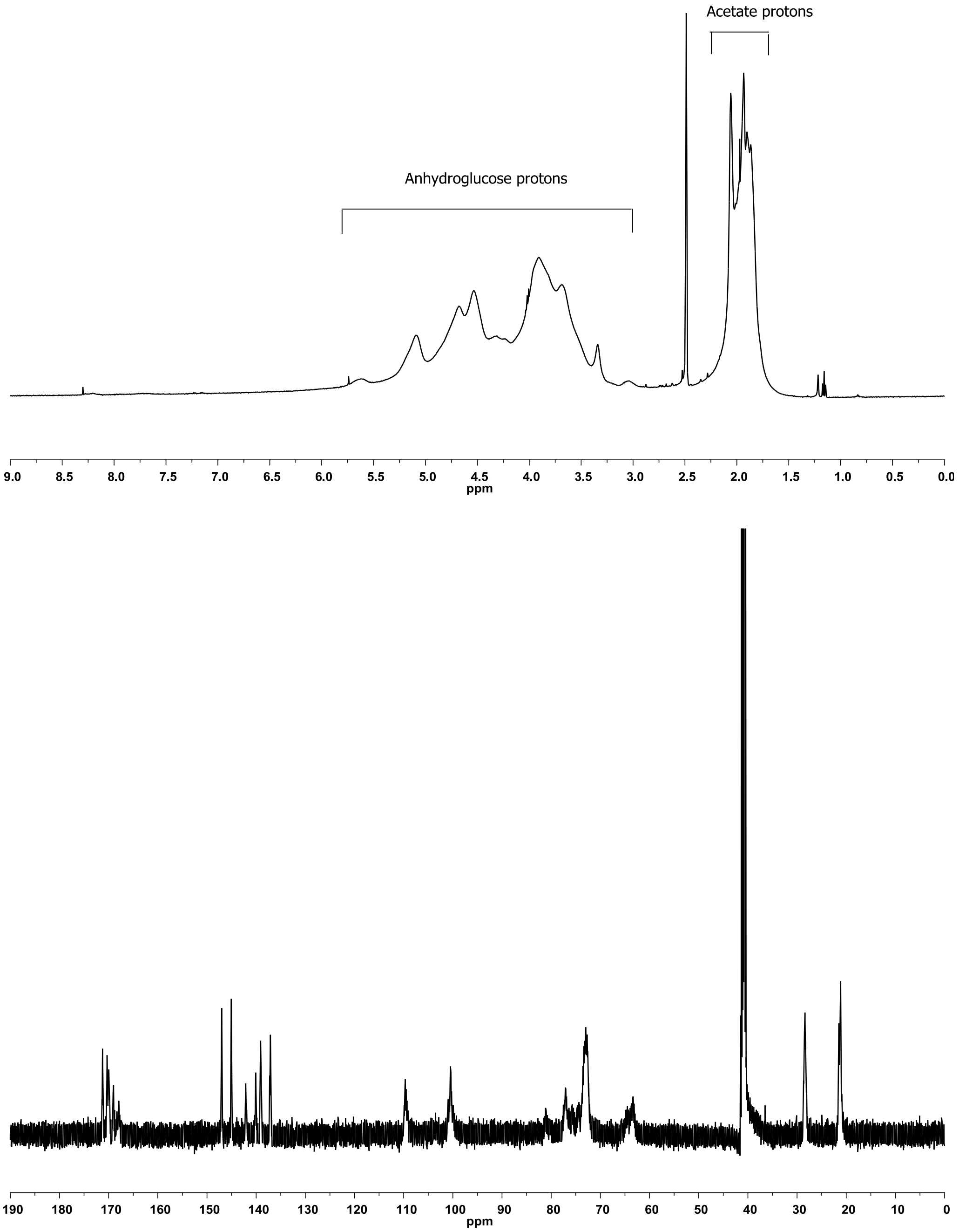

Figure S5. ^{1}H NMR (**top**) and ^{13}C NMR (**bottom**) spectra of F5Ph-CA.

E. Phenylacetyl-Functionalized Cellulose Acetate (Ph-CA)

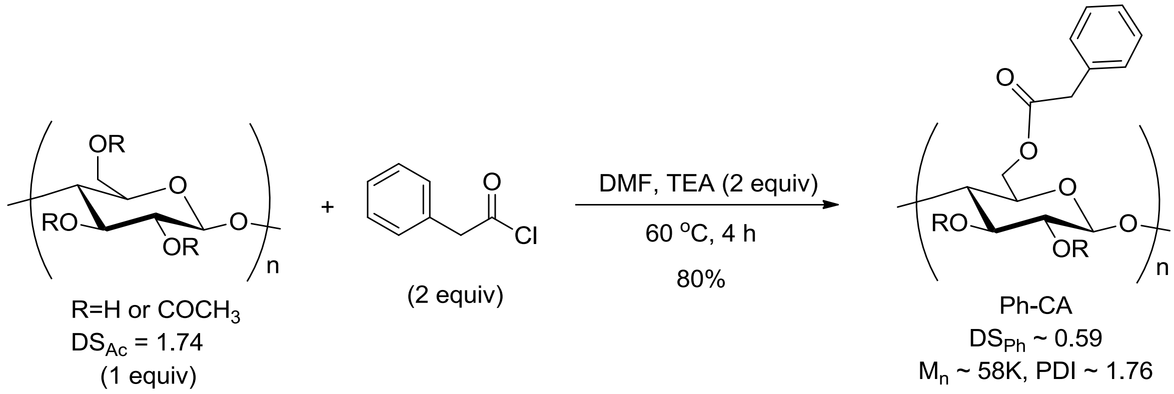

Phenylacetyl-functionalized cellulose acetate (Ph-CA) was prepared by the same procedure for F5Ph-CA. The degree of substitution of Ph selector (DS_{Ph}) was 0.59 from elemental analysis, and Ph-CA was soluble in acetone, THF, DMSO, and DMF. M_n: 58 KDa, M_w: 102 KDa, PDI: 1.76; Elemental analysis: C 55.97, H 5.17, N 0.26.

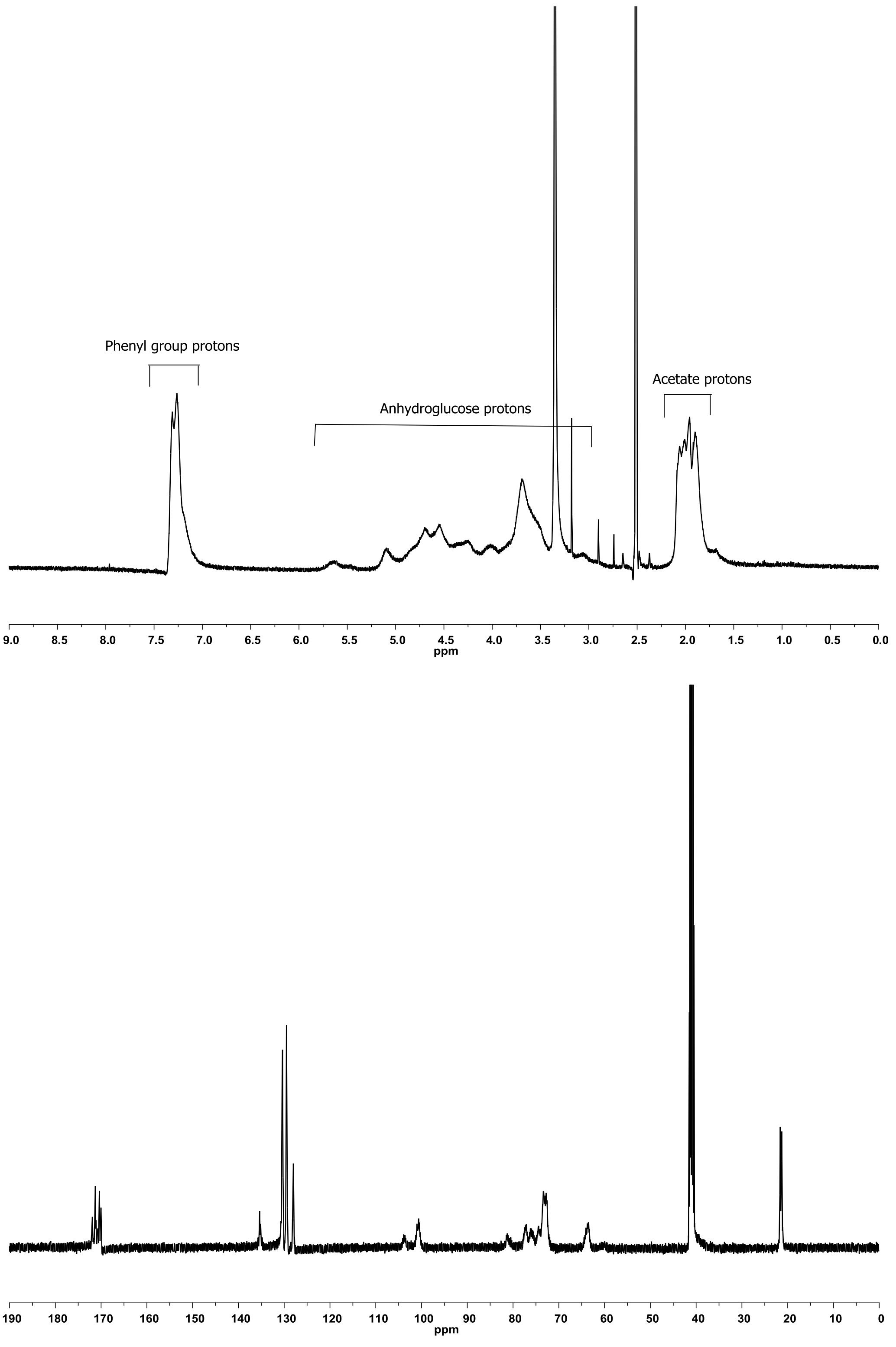

Figure S6. ^{1}H NMR (**top**) and ^{13}C NMR (**bottom**) spectra of Ph-CA.

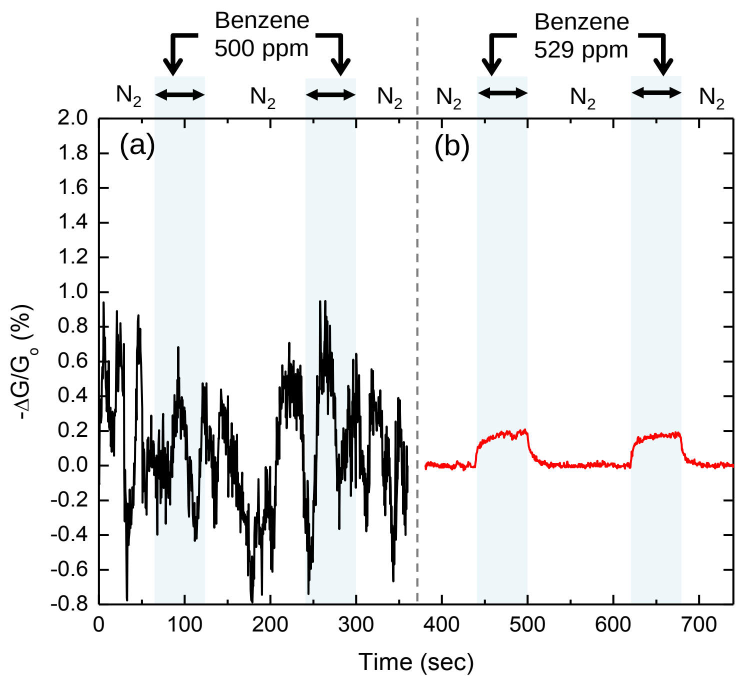

Figure S7. Normalized conductance changes [$-\Delta G/G_o$ (%)] of the device prepared from (**a**) the premixed F5Ph-CA polymer and SWCNTs and (**b**) the integrated F5Ph-CA concentrator/SWCNT system (F5Ph-CA/SWCNT) when exposed to benzene vapor.

Calculation of the Detection Limit

The detection limit of a sensor was calculated by extrapolating the linear calibration curve ($-\Delta G/G_o$ vs concentration) when the signal equals three times the noise [4]. The noise level of a sensor can be calculated from the root-mean square deviation of the data at the baseline. After a fifth-order polynominal fitting of the curve of the normalized conductance change as a function of concentration using Origin 8.0, the variance ($V_{\chi 2}$) was calculated from the residual (χ) between 10 data points at the baseline and curve fitting results by the following equation:

$$V_{\chi^2} = \sum (y_i - y)^2$$

where y_i is the measured data point and y is the corresponding value calculated from the curve-fitting equation. The average noise level (rms_{noise}) was calculated as follows:

$$rms_{noise} = \sqrt{\frac{V_{\chi^2}}{10}}$$

The detection limit (LOD at signal-to-noise = 3) was calculated as follows:

$$LOD = 3\frac{rms_{noise}}{slope}$$

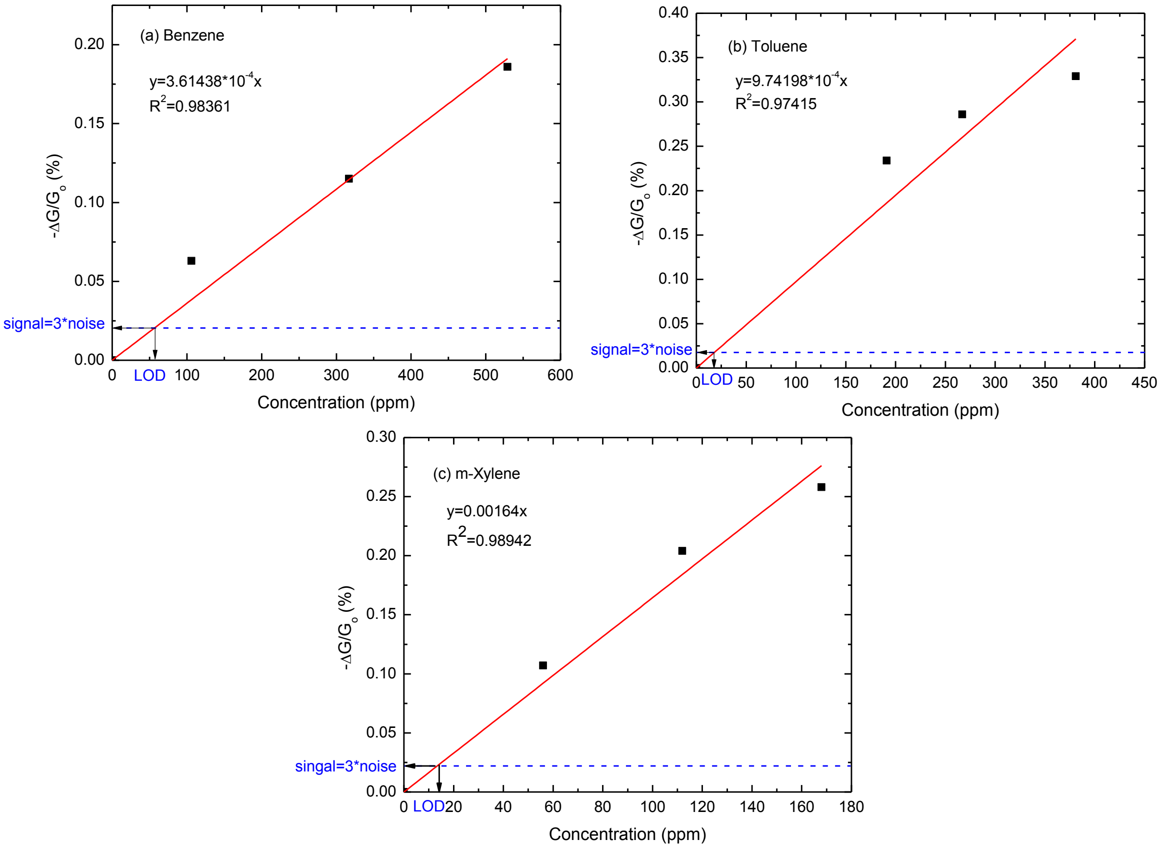

Figure S8. Normalized conductance changes of the F5Ph-CA/SWCNT sensor as a function of concentration of (**a**) benzene, (**b**) toluene, and (**c**) *m*-xylene vapors showing the limit of detection (LOD) where the signal equals three times the noise.

Influence of Humidity on Sensor Performance

The performance of the F5Ph-CA/SWCNT sensing system at different humidity levels was investigated by measuring the conductivity change of the system when exposed to benzene with the humidity levels of 8.5% and 66%. For this study, we used compressed air with a humidity level of 8.5% as a carrier gas. High humidity gas stream was achieved by bubbling compressed air (humidity: 8.5%) through water and mixed with the saturated benzene vapor to generate benzene vapor with a humidity level of 66%. Relative humidity was measured using a TM-183 Temperature/Humidity Meter (TENMARS Electronics Co., LTD).

Figure 9S shows the normalized conductivity change [$-\Delta G/G_o$ (%)] of the integrated F5Ph-CA concentrator/SWCNT system (F5Ph-CA/SWCNT) when exposed to benzene of varying concentrations in the presence of air with humidity levels of 8.5% and 66%. The F5Ph-CA/SWCNT system detected benzene vapor of 529 ppm in the air with humidity of 8.5%, which was not as strongly as in dry nitrogen as a carrier gas. The responses of the sensors upon exposure to benzene vapor of 529 ppm in the presence of air with humidity level of 8.5% decreased almost 98%, compared to the responses toward benzene vapor of 529 ppm in dry nitrogen. Under high humidity condition (66%), no response was observed at any benzene concentration.

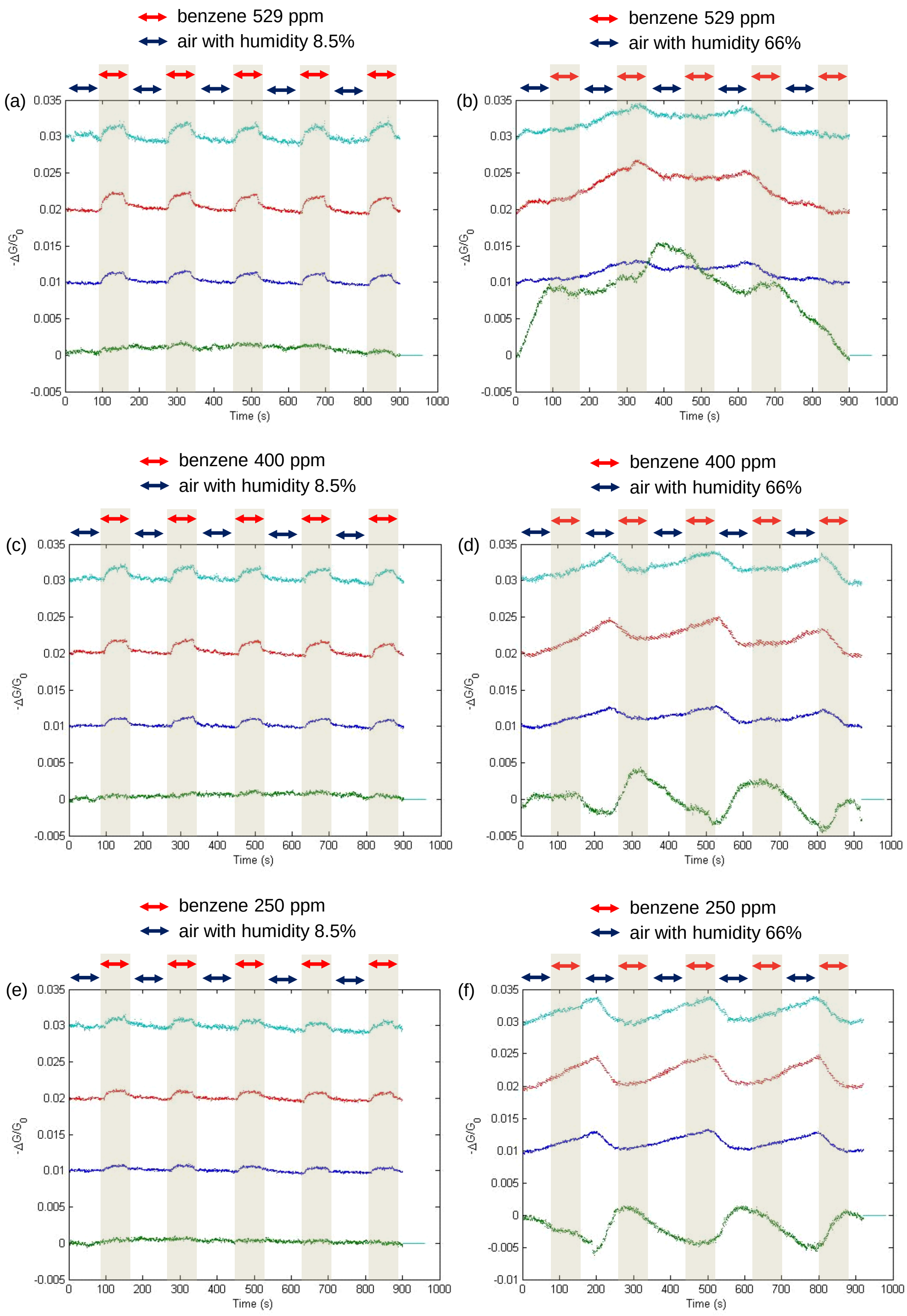

Figure S9. Normalized conductance changes [$-\Delta G/G_o$ (%)] of the integrated F5Ph-CA concentrator/SWCNT system (F5Ph-CA/SWCNT) when exposed to benzene in the presence of humid air: (**a**) benzene of 529 ppm with a humidity level of 8.5%; (**b**) benzene of 529 ppm with a humidity level of 66%; (**c**) benzene of 400 ppm with a humidity level of 8.5%; (**d**) benzene of 400 ppm with a humidity level of 66%; (**e**) benzene of 250 ppm with a humidity level of 8.5%; and (**f**) benzene of 250 ppm with a humidity level of 66%. In all figures, the bottom trace (green) was the response of the pristine SWCNT sensor, while the responses of a triplet of the F5Ph-CA/SWCNT were shown to be blue, red, and aqua.

References

1. Lobez, J.M.; Swager, T.M. Radiation detection: resistivity responses in functional poly(olefin sulfone)/ carbon nanotube composites. *Angew. Chem. Int. Ed.* **2010**, *49*, 95–98.
2. Meadows, E.S.; Wall, S.L.D.; Barbour, L.J.; Gokel, G.W. Alkali metal cation-π interactions observed by using a Lariat ether model system. *J. Am. Chem. Soc.* **2001**, *123*, 3092–3107.
3. Shey, J.; Holtman, K.M.; Wong, R.Y.; Gregorski, K.S.; Klamczynski, A.P.; Orts, W.J.; Glenn, G.M.; Imam, S.H. The azidation of starch. *Carbohydr. Polym.* **2006**, *65*, 529–534.
4. Li, J.; Lu, Y.; Ye, Q.; Cinke, M.; Han, J.; Meyyappan, M. Carbon nanotube sensors for gas and organic vapor detection. *Nano Lett.* **2003**, *3*, 929–933.

© 2016 by the authors; licensee MDPI, Basel, Switzerland. This article is an open access article distributed under the terms and conditions of the Creative Commons by Attribution (CC-BY) license (http://creativecommons.org/licenses/by/4.0/).
